# Exogenous Hormones Affect the Corm Expansion of *Sagittaria trifolia* in Hydroponic Conditions

**DOI:** 10.3390/plants15131984

**Published:** 2026-06-26

**Authors:** Jiexia Liu, Ziqi Xiong, Enjiao Li, Jiahui Shen, Enming Liu, Wenwen Ding, Liangjun Li

**Affiliations:** 1 College of Horticulture and Landscape Architecture, Yangzhou University, Yangzhou 225009, China; 2 Key Laboratory of Biobreeding for Specialty Horticultural Crops of Jiangsu Province, Joint International Research Laboratory of Agriculture and Agri-Product Safety of Ministry of Education of China, Yangzhou University, Yangzhou 225009, China

**Keywords:** corm expansion, hydroponic, plant hormone, arrowhead

## Abstract

The tip of the underground stolon of arrowhead (*Sagittaria trifolia*) can perceive rhizosphere mechanical resistance and promote corm expansion by regulating the biosynthesis and accumulation of plant hormones. However, corm development of *S. trifolia* is affected under hydroponic conditions with low mechanical resistance. This study aimed to promote the formation and expansion of *S. trifolia* corms under hydroponic conditions and explore its potential regulatory mechanism through exogenous hormone application and transcriptome analysis. Exogenous ABA, JA, and SA was applied to hydroponic *S. trifolia*, and the key candidate genes regulating corm expansion were identified by transcriptome assay. The results showed that both MeJA and ABA could promote the corm expansion under hydroponic conditions, while SA inhibited corm development at the early stage of treatment and induced a large number of stolons to emerge and branch from the underground part of *S. trifolia* at the later stage. Transcriptome analysis showed that the key genes related to ethylene, auxin and cytokinin, namely *StriChr1G051240* (*EIN3*), *StriChr3G133510* (*EIN3*), *StriChr2G094080* (*ARF*), *StriChr3G125830* (*ARR*), *StriChr4G170530* (*ARF*), and *StriChr5G184020* (*ARF*), were all differentially expressed in expanded *S. trifolia* corms that were induced by ABA treatment and high rhizosphere mechanical resistance. This finding indicated that ABA may act together with ethylene, auxin and cytokinin by regulating the expression of *EIN3*, *ARR* and *ARF* genes to promote the corm formation, in the condition of insufficient mechanical resistance. Current study provides a theoretical basis for elucidating the hormonal regulatory of *S. trifolia* corm expansion and improving the delayed corm expansion problem under hydroponic cultivation.

## 1. Introduction

*Arrowhead (Sagittaria trifolia*) is a perennial herb belonging to the genus *Sagittaria* within the family Alismataceae. *S. trifolia* is an aquatic vegetable with high economic value, which grows in paddy fields and requires a large water supply throughout its growth period [1]. The plant is tall and erect, with distinctive arrow-shaped leaves as its key morphological characteristic. In China, it is widely cultivated in Jiangsu, Guangxi, and Yunnan provinces. The edible product organ is the corm formed by the expanded tip of the underground stolon, with significant nutritional and medicinal value [2,3,4,5].

Hydroponics is a soilless cultivation method for plants, also known as nutrient solution culture. Its core principle is to immerse plant roots directly in a nutrient solution, replacing soil by providing water, nutrients and other growth factors, enabling plants to grow normally and form yield [6]. This cultivation mode has significant advantages such as water and fertilizer saving, fast growth, high yield, freedom from soil-borne diseases, clean products and annual intensive production, making it an important technical direction of modern efficient protected agriculture [7,8]. At present, it has been widely practiced in small-sized leafy vegetables, including lettuce [9], celery [10], coriander [11] and spinach [12], but is less applied in plants with underground tissues as the product organs. The existing case of hydroponics for underground product organs is the production of potato mini-tubers [13]. Previous studies on hydroponics of *S. trifolia* also found that the expansion of underground corms was delayed and the corms were smaller under the condition of lacking soil mechanical resistance [14]. It is worth exploring how to improve the formation and development of product organs in hydroponic root and tuber crops through the application of exogenous substances.

Plant hormones play a crucial role in regulating crop growth and development, including controlling cell division and elongation, modulating plant immunity, and enhancing stress tolerance [15,16,17,18]. In the study on mechanical resistance-mediated corm expansion of *S. trifolia*, it was found that the accumulation levels of ABA (abscisic acid), JA (jasmonic acid), and SA (salicylic acid) at the stolon tips of hydroponically grown *S. trifolia* with delayed corm formation were significantly different from those of sand-cultured *S. trifolia* that formed corms normally, indicating that these three plant hormones may play important roles in regulating corm development in hydroponic *S. trifolia* [14]. Exogenous application of plant hormones affects crop growth. Reports have demonstrated that ABA and GA treatments can regulate the expansion of underground storage organs in *Dioscorea* crops [19]. Early studies have found that the application of JA inhibitors inhibits the tuber formation of potato [20], the antagonism between ABA and GA regulates the corm development of *Gladiolus hybridus* [21], and the simultaneous application of JA and SA induces the formation of potato tubers in vitro [22]. The regulatory effects of these exogenous plant hormones on the development of hydroponic *S. trifolia* corms are not yet clear.

As the main edible organ of *S. trifolia*, the development of the underground corm is critical for determining its commercial value and yield. Based on the conclusion of Li et al. 2023 [14], in this study, the effects of exogenous hormones ABA, MeJA, and SA on the corm expansion in *S. trifolia* plants grown under hydroponic conditions (low mechanical resistance) were investigated. Combined with high-throughput sequencing, we further explored the roles of plant hormone signals in regulating corm expansion in response to missing mechanical resistance. This finding will fill the research gap concerning plant hormones’ functions in the establishment and application of *S. trifolia* hydroponic systems.

## 2. Results

### 2.1. Effects of Exogenous Hormones on Corm Expansion of Hydroponic S. trifolia

**Figure 1:**
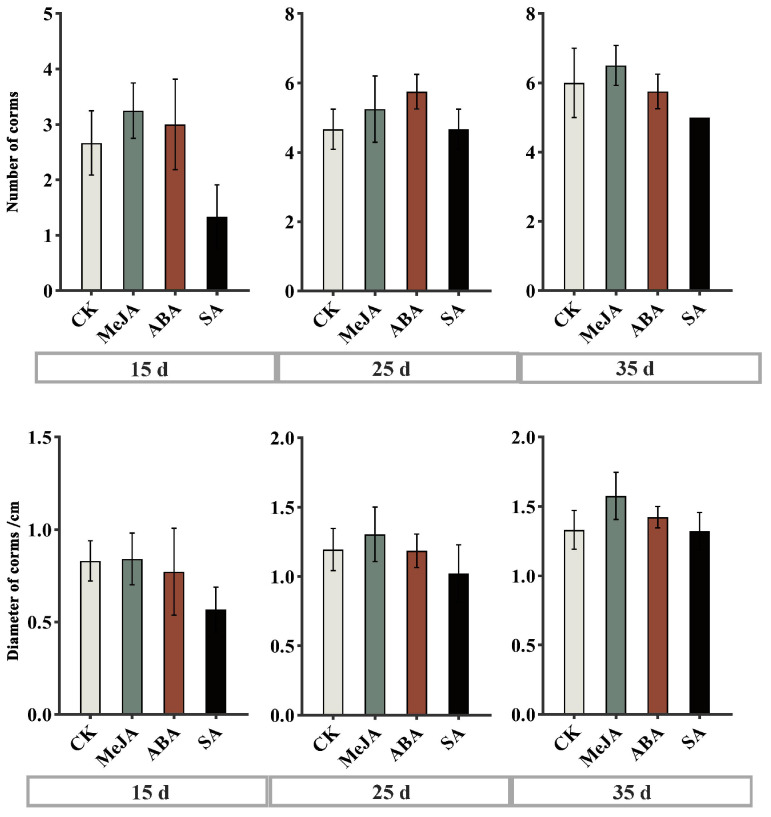
Effects of different hormone treatments on the number and diameter of corms in hydroponic arrowhead plants.

The corm development of hydroponic *S. trifolia* in ABA-, MeJA-, and SA-treated groups and the control is shown in Figure 1. After 15 days of hormone application, a small number of initially expanded corms were observed in all hydroponic groups. The number of expanded corms of *S. trifolia* treated with MeJA and ABA increased, indicating that MeJA and ABA could promote the formation of *S. trifolia* corms in hydroponic conditions. The number of emerged corms (only count the corms on the creeping stems that originated from the root base) in the SA-treated group was less than that of the control group. After 25 days of growth, the number of expanded corms in the hydroponic *S. trifolia* treated with MeJA and ABA remains higher than that of the control group; the expanded corm numbers in the SA-treated group were similar to those of the control group. The differences in generated corm numbers gradually diminish with increased growth time. The above results indicated that the application of MeJA and ABA has a promoting effect on the early stage of corm development of *S. trifolia* in hydroponic conditions, while the result in the SA group was opposite.

Among MeJA-, ABA-, and SA-treated groups, the diameter of the hydroponic *S. trifolia* corms in the SA-treated group was smaller than the control at 15 and 35 days after treatment. After 35 days, it was found that the corm diameters of *S. trifolia* grown in MeJA and ABA were greater than those of the control, indicating that the application of MeJA and ABA could promote the corm expansion of hydroponic *S. trifolia*.

**Figure 2:**
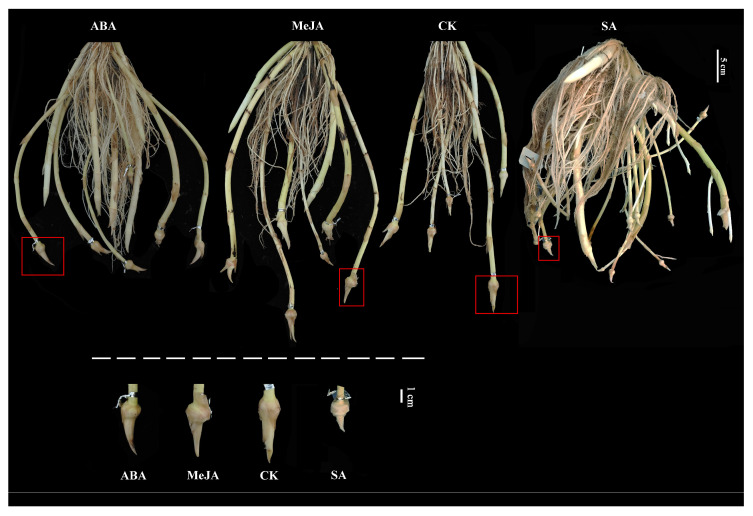
Growth status of *S. trifolia* underground in hydroponics at 35 d after hormone treatment. The dotted box revealed the representative corms of hydroponic arrowhead (35 days after treatment) under each treatment condition.

After 35 days of exogenous hormone treatment, the growth status of the *S. trifolia* underground part in hydroponic conditions was shown in Figure 2. The *S. trifolia* plants from all hydroponic groups had formed a large number of corms at this point. In the late growth stage of SA-treated hydroponic *S. trifolia* (35 days after SA application), the underground root system grew vigorously, and a large number of stolons emerged; branched stolons were produced from the primary stolons, and new corms were formed from the branched stolons, showing the representative phenotype of the underground of SA-treated *S. trifolia*. Among these hormone-treated groups, the size of the hydroponic *S. trifolia* corms treated by MeJA and ABA was larger, while the corms of the SA-treated group were smaller. These findings demonstrated that exogenous addition of ABA and MeJA promoted the expansion of *S. trifolia* corms in hydroponic conditions.

### 2.2. Transcriptome Analysis

Our previous work described that the development of corms in hydroponic *S. trifolia* was delayed due to the absence of rhizosphere mechanical resistance compared to sand cultivation, and the stolon tips of hydroponic *S. trifolia* harboring unexpanded corms, as expected, contained higher JA accumulation and lower ABA content [14]. Combined with the results of this study, it is speculated that ABA plays an important role in promoting the *S. trifolia* corm development under hydroponic conditions lacking rhizosphere mechanical resistance. Therefore, we further performed transcriptome sequencing using ABA-treated tissues of hydroponic *S. trifolia* plants to explore the underlying molecular regulatory mechanism. Transcriptome sequencing, assembly, and functional annotation were performed. The statistics of sequencing data were listed in Table S1. The effective reads of each sample reached more than 39,400,308, and the number of effective bases was not less than 5,807,995,937. The percentage of Q20 and Q30 were 98% and 95%, respectively; the GC content was 51%, suggesting the quality control effect of all data was great and could be used for subsequent analysis.

The DEGs were identified from the comparison of CKexp vs. ABAexp, CKunexp vs. ABAexp, and CKunexp vs. CKexp, respectively (Table 1). A total of 2943 DEGs were screened by comparing the transcriptome data of CKunexp and CKexp, and the number of upregulated and downregulated DEGs was 1274 and 1669, respectively. In the CKexp vs. ABAexp group, 571 DEGs were upregulated, and 1806 were downregulated. In the CKunexp vs. ABAexp group, 1284 DEGs were upregulated, and 2408 were downregulated.

### 2.3. KEGG Enrichment Analysis of DEGs Identified form Hydroponic Arrowhead

**Figure 3:**
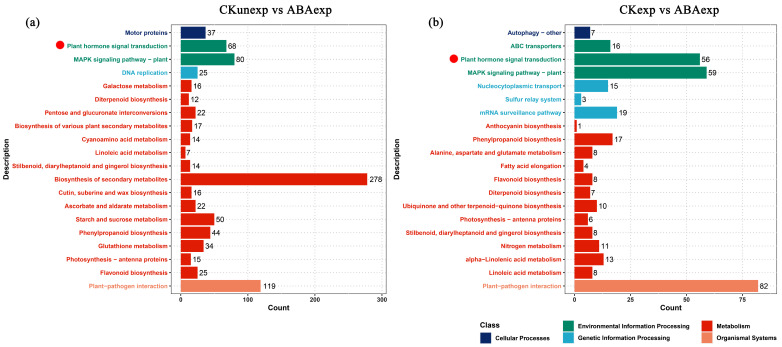
KEGG enrichment analysis of DEGs. (**a**) Top 20 enriched KEGG pathways of DEGs idetified from the comprision of CKunexp vs. ABAexp. (**b**) Top 20 enriched KEGG pathways of DEGs idetified from the comprision of CKexp vs. ABAexp. The red circle indicates the plant hormone signal transduction pathway of interest in this study.

Identified DEGs were blasted to the KEGG (Kyoto Encyclopedia of Genes and Genomes) database, and the top 20 KEGG pathways with the highest number of DEGs enrichment are illustrated in Figure 3. In the DEGs identified by CKunexp vs. ABAexp and CKexp vs. ABAexp comparisons, 68 and 56 genes were enriched in the pathway of plant hormone signal transduction, respectively, suggesting that hormone signaling also plays an important role in the process of exogenous ABA-induced *S. trifolia* corm expansion in hydroponic conditions. In addition, in the comparison of CKunexp vs. ABAexp, it was found that many DEGs were enriched in the starch and sucrose metabolism pathway. However, in the comparison results of CKexp vs. ABAexp, which were collected from the expanded corms, DEGs were not significantly enriched in this pathway. It is speculated that the accumulation of starch and other metabolites may contribute to the expansion of hydroponic *S. trifolia* corms.

### 2.4. The Expression Differences in Genes Related to Plant Hormone Signal Transduction

**Figure 4:**
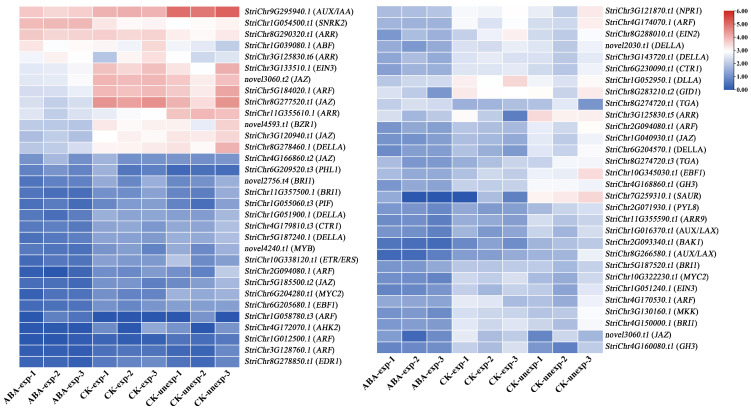
The expression heatmap of hormone-related DEGs in arrowhead among the CKunexp, CKexp and ABAexp groups.

To further explore how exogenous ABA signaling can promote the expansion of hydroponic *S. trifolia* corms, the DEGs associated with hormone signal transduction enriched in the two groups of CKexp vs. ABAexp and CKunexp vs. ABAexp were integrated, and then the common DEGs in the CKunexp vs. CKexp were removed. The key DEGs that may be involved in response to ABA signaling to modulate the corm expansion of *S. trifolia* were obtained. The expression of these DEGs was shown in Figure 4. The results showed that the positive regulator of the ABA signaling pathway, *StriChr1G054500.t1* (*SNRK2*), was significantly upregulated in the corms of *S. trifolia* in the ABAexp group. *StriChr3G133510.1* (*EIN3*), a key regulator of the ETH signaling pathway, was significantly downregulated in the ABAexp group, suggesting that ETH may be involved in the response to exogenous ABA signaling. In addition, many genes related to auxin signal transduction were screened, such as the *StriChr8G266580.1* (Auxin Influx Carrier 1 gene, *AUX/LAX*), which was highly expressed in all samples; the expression of *StriChr5G184020.1*, *StriChr4G174070.1*, and *StriChr2G094080.1,* belonging to auxin response factor (ARF) family genes, was downregulated in ABAexp. These results suggested that exogenous ABA application may negatively regulate auxin response in hydroponic *S. trifolia* corms. In addition, other hormone signaling pathway-related genes were downregulated after ABA treatment, including *StriChr8G278460.1* (*DELLA*), which encodes a GA negative regulator, and *StriChr8G277520.t1* (*JAZ*), which is a member of TIFY family involved in JA metabolism. *novel4593.t1* (*BZR1*), an important inhibitor of the brassinosteroid (BR) signaling pathway, and *StriChr3G121870.t1* (*NPR1*), a gene encoding the SA receptor, also exhibited decreased expression after ABA treatment. The differences in the expression levels of related genes in these hormone signaling pathways suggested that complex signal transduction involving multiple hormones may occur in hydroponic corms in response to exogenous ABA application. Various hormones work together to regulate the expansion of *S. trifolia* corms induced by ABA application.

### 2.5. Transcriptome Cross-Analysis and RT-qPCR Validation

Transcription factors are widely involved in plant growth regulation and stress response. To further screen the key hormone-related regulators involved in exogenous ABA-promoted corm expansion of hydroponic *S. trifolia* under the condition of lacking rhizosphere mechanical resistance, we performed a cross-analysis between DEGs induced by exogenous ABA and those identified from the comparison of sand-cultured and hydroponic *S. trifolia*. A total of six candidate key transcription factors were screened, including *StriChr1G051240* (*EIN3*), *StriChr3G133510* (*EIN3*), *StriChr2G094080* (*ARF*), *StriChr3G125830* (*ARR*), *StriChr4G170530* (*ARF*), and *StriChr5G184020* (*ARF*), respectively (Table S2).

**Figure 5:**
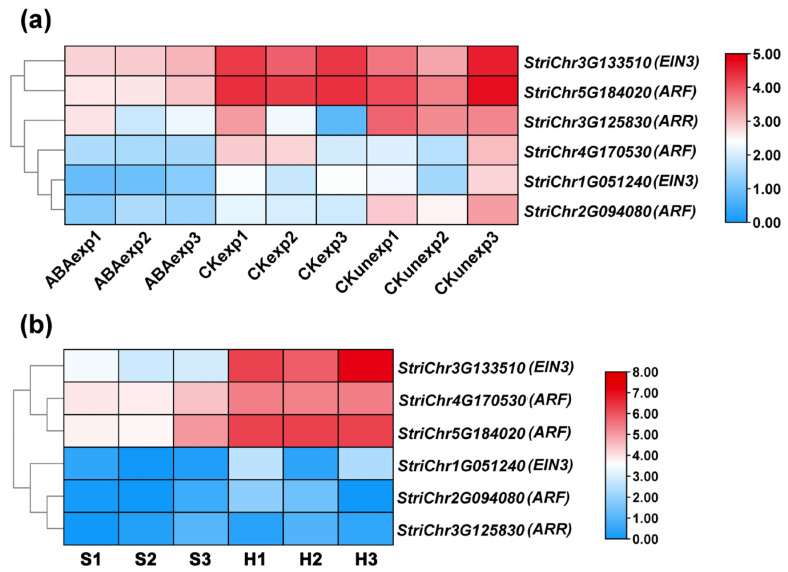
Expression heatmap of candidate transcription factor genes identified from cross-analysis. (**a**) Expression heatmap of six genes in expanded corms, unexpanded stolon tip between ABA-treated and control group. (**b**) Expression heatmap of six genes in expanded corms, unexpanded stolon tip between sand-cultured and hydroponic group.

The expression of six candidate transcription factor genes in all sequencing samples was analyzed (Figure 5). Among them, *EIN3* is a key regulator of ETH signaling, and ARF transcription factor is an auxin response factor. The genes encoding the EIN3 transcription factors, *StriChr1G051240* (*EIN3*) and *StriChr3G133510* (*EIN3*), were significantly downregulated in sand-cultured (S) *S. trifolia* corms with high mechanical resistance and the ABA-treated hydroponic *S. trifolia* corms. The expression of several *ARF* genes involved in the response to the auxin signaling was downregulated in both ABAexp and sand-cultured corms. The up-/downregulation results of six transcription factor genes in sand-cultured *S. trifolia* corms with higher mechanical resistance were similar to those in expanded corms induced by ABA application. These six candidate genes were closely related to the metabolism of endogenous ETH, auxin, and CTK in plants, respectively, indicating that mechanical resistance and ABA signaling may affect the metabolism of endogenous hormones such as ETH, auxin, and CTK, which synergistically promote the expansion of *S. trifolia* corms.

**Figure 6:**
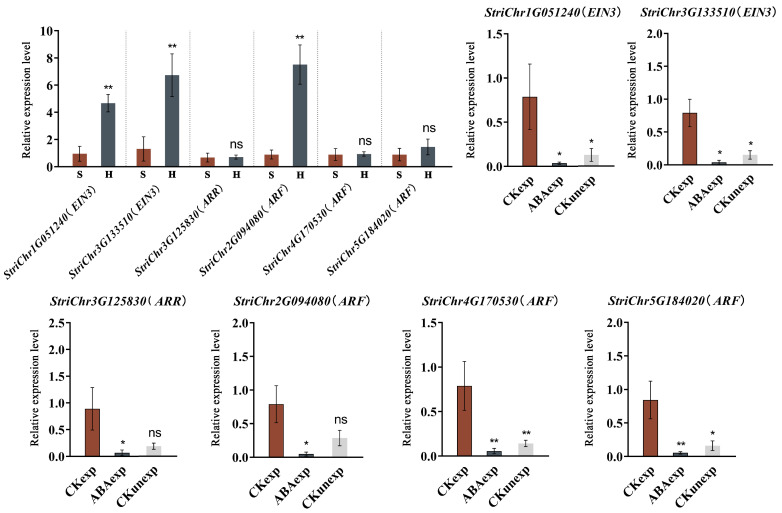
RT-qPCR verified the relative expression levels of six transcription factors. S represents the sand-cultured group, H represents hydroponics; ns represents no significance, * *p* < 0.05, ** *p* < 0.01.

The expression of the identified genes in all samples was further verified by real-time quantitative PCR (RT-qPCR) (Figure 6). The expression of three transcription factor genes, *StriChr1G051240* (*EIN3*) and *StriChr3G133510* (*EIN3*) associated with ETH metabolism and *StriChr2G094080* (*ARF*) associated with auxin metabolism, was significantly downregulated in the expanded corms of the sand-cultured group, when comparing the effects of high/low mechanical resistance on the expansion of *S. trifolia* corms. This result was consistent with the transcriptome data. Compared with the CKexp group, these six genes were significantly downregulated in the expanded corms of the ABAexp-hydroponic group. Based on the exploratory analysis results from different culture systems, it is preliminarily inferred that both exogenous ABA and high mechanical resistance in the rhizosphere can regulate the expression of *EIN3* and *ARF*, and participate in the development of *S. trifolia* corms by interacting with endogenous ethylene and auxin signaling. ABA treatment may mimic the effects of rhizosphere mechanical resistance, thereby regulating corm expansion.

## 3. Discussion

The tip of the underground stolon of *S. trifolia* could perceive rhizosphere mechanical resistance to modulate the biosynthesis and accumulation of plant hormones, thereby promoting the formation and expansion of its corms [14]. In the production of hydroponic *S. trifolia*, it is urgent to overcome the problem that corm formation is significantly delayed under hydroponic conditions with low mechanical resistance. Accordingly, in this study, we attempted to exogenously apply plant hormones (including ABA, JA and SA) that showed significant changes in accumulation under high rhizosphere mechanical resistance to hydroponic *S. trifolia*, aiming to offset the adverse effects caused by insufficient mechanical resistance [14]. Previous studies have demonstrated that rhizome mechanical signals are first perceived by underground tissues and then transmitted via phytohormone signaling cascades to regulate organ development [23]. Here, key candidate genes regulating corm expansion in response to mechanical resistance were explored through transcriptome comparison and cross-analysis, which will lay a foundation for the future realization of hydroponic *S. trifolia* production via breeding and other strategies.

MeJA functions as a key regulator in plant growth and development, stress responses, and secondary metabolism [24]. Here, we found that MeJA application promoted the expansion of corms in hydroponic conditions. Exogenous application of ABA significantly promoted corm expansion in hydroponically grown *S. trifolia*, suggesting that ABA plays a positive role in this process. Starch was the major component of *S. trifolia* corms [25]. Previous studies have also reported that exogenous ABA could enhance starch accumulation and promote the expansion of underground storage organs [26]. ABA induced the upregulated expression of *NnABI4*, and *NnABI4* promoted the expression of *NnSS1* by directly binding to its promoter, thereby enhancing starch accumulation in lotus rhizomes [26]. In cassava, MeSnRK2.3 (an ABA-dependent kinase) interacts with MebHLH68 to mediate ABA-regulated starch synthesis. MebHLH68 directly binds the promoters of *MeSUS1*, *MeGBSSIa* and *MeSBE2.4* to activate their transcription, and *MebHLH68* overexpression increased root starch accumulation and plant growth [27]. However, the promoting effect of ABA on hydroponic *S. trifolia* was more pronounced at the early treatment stage. Soil compaction induces the expression of endogenous ABA biosynthesis genes and an increase in ABA concentration, which in turn triggers radial expansion of root cortex cells [28]. Root tissues continuously perceive rhizosphere mechanical resistance, which drives persistent, dynamically balanced endogenous hormone fluctuations. A single dose of exogenous ABA added to the nutrient solution triggers immediate phenotypic changes in underground tissues upon rapid uptake; subsequently, ABA concentrations decrease daily owing to microbial degradation and plant metabolic consumption. Therefore, its facilitative effect on *S. trifolia* corm expansion may diminish over time. SA inhibited corm expansion at the early application stage. At the later stage, a large number of stolons emerged from the underground part of *S. trifolia*, as well as new stolons further branched from these stolons. Relevant studies have shown that the impact of SA on lateral root formation is dose-dependent, and a low concentration of SA increases lateral root number in plant tissue cultures of the Catharanthus roseus hairy root line obtained from *Agrobacterium rhizogenes*-infected leaves [29,30]. Therefore, we speculate that this phenomenon may be related to the induction of lateral root formation by SA. In previous studies, unlike exogenous hormone application, which affects the overall underground growth of hydroponic *S. trifolia*, mechanical signals may locally activate SA biosynthesis only at the meristematic sites of stolons. SA accumulates specifically at the corm differentiation region of stolon tips to directionally regulate corm expansion, with barely any influence on lateral stolon initiation [14].

To further explain the regulatory role of hormone signaling in the corm expansion of hydroponic *S. trifolia*, high-throughput sequencing technology was used to complete the transcriptome sequencing of the *S. trifolia* of the ABA-treated group and the no-hormone-added group. In the constructed expression profile of DEGs, the expression levels of multiple genes related to hormone signal transduction in the corms of the ABAexp group were downregulated, including multiple genes from EIN3, ARF, and TIFY families. Studies have demonstrated that ETH and ABA exert antagonistic modulation in plants [31]. TIFY and MYC2 can be involved in responding to multiple hormonal signals [32]. The expression of genes in multiple ARF families was downregulated in the corms of the ABAexp group. As key components of hormone signaling, ARF proteins can integrate signals from multiple phytohormones and mediate subsequent cell growth and organ differentiation [33]. This finding suggested that there may also be crosstalk between auxin, JA, and ABA, similar to other crops in the regulation of corm development [34,35]. This preliminary hypothesis requires further experimental validation. DEGs involved in BR and SA metabolism have also been identified in ABA-induced corm development in hydroponic conditions. Published studies have reported on the interaction between ABA and BRs and SA [36,37]. These hormones regulate plant growth and development through the interaction between key genes in their signaling pathways [38]. We inferred that the crosstalk between multiple hormonal signaling pathways is also involved in regulating corm formation of *S. trifolia*.

Cell number and volume are also important factors contributing to corm expansion in *S. trifolia*. The roles of auxin and CTK in plant growth are achieved by regulating cell proliferation and cell expansion [39,40]. Based on transcriptome analysis, we preliminarily identified differentially expressed transcription factor genes that are associated with rhizosphere mechanical resistance-promoted corm expansion in sand-cultured *S. trifolia* and also participate in ABA-induced corm formation in hydroponic *S. trifolia*. These include six genes encoding EIN3, ARR, and ARF. ARR is a positive regulator of CTK synthesis [41], ARF is an auxin response factor [42], and EIN3 is a key transcription factor for ETH signal transduction [43]. ARR family transcription factors are involved in plant abiotic stress responses. It has also been reported that ARR can mediate signal crosstalk of CTK and ETH [44]. ETH and ABA typically work together in plant growth and development, and have been widely reported. In *Populus trichocarpa*, several *PtrARF* genes, with at least one *cis*-element related to ABA response, responded differently to exogenous ABA treatment [45]. The coordinated roles of ethylene and ABA in plant growth and development have been widely documented [46,47,48]. The cross-analysis of this finding and previous sand-culture-versus-hydroponics findings, as an exploratory screening, identified the candidate genes and regulatory pathways with potential relevance to corm expansion triggered by mechanical stimuli and ABA signals.

In conclusion, exogenous ABA and MeJA promoted corm expansion under hydroponic conditions, while SA delayed early corm expansion and altered stolon development. Transcriptome and RT-qPCR analyses suggested that ABA-induced corm expansion is associated with changes in hormone-signaling transcription factors, including *EIN3*, *ARF*, and *ARR* genes. Nevertheless, exogenous hormones cannot recapitulate the endogenous homeostasis induced by mechanical signals, and phenotypes generated by exogenous hormone spraying may show deviations. In follow-up studies, we will further verify endogenous hormone regulatory pathways using gene-edited and overexpressed materials to dissect the molecular mechanisms through which hormones regulate crop phenotypes.

## 4. Materials and Methods

### 4.1. Plant Treatment

The *S. trifolia* cv. ‘Baimati’ plants were used as experimental materials. The complete corms with full terminal buds were selected, cleaned, and put into the plant pot containing municipal water, and then placed in a greenhouse with sufficient light for seedling cultivation. The seedling stage was about 30 days. When the *S. trifolia* seedlings grew to 20 cm in height, healthy and well-proportioned *S. trifolia* seedlings were selected and transplanted into hydroponic pots for cultivation. The supply of nutrient solution was as described by Li et al. 2023 [14].

At 30 days after hydroponic planting, when 2~3 stolons had emerged, the *S. trifolia* plants were divided into 4 groups. Each group consisted of 12 *S. trifolia* plants, with 2 planted in each pot (diameter: 50 cm, height: 38 cm). One group was the control, and the other three groups had ABA, MeJA, and SA added to the hydroponic nutrient solution, respectively. Referring to the concentration screening results of exogenous ABA, MeJA and SA in various crops from previous studies, 10 mg/L ABA, 1 μmol/L MeJA and 1 mg/L SA were applied as treatments in this experiment [49,50]. ABA, MeJA, and SA were dissolved in absolute ethanol to prepare stock solutions of 50 mg/mL, 50 μmol/L, and 1 mg/mL, respectively. These stock solutions were then diluted 5000-fold to obtain final concentrations of 10 mg/L, 1 μmol/L, and 1 mg/L. Then, 15 days after hormone application, fertilizer was supplemented as previously described [14], without supplementary addition of the corresponding hormones.

### 4.2. Underground Growth Monitoring

After the application of MeJA, ABA, and SA, the number and diameter of the *S. trifolia* corms were measured on the 15th, 25th, and 35th days of hormone application, respectively. Three biological replicates of plants from each treatment group were randomly selected for measurement. The measurements at each time point were performed on different biological replicate plants.

### 4.3. Sampling Collection, RNA Extraction, and Transcriptome Sequencing

On the 15th day of hormone addition, the expanded corms of hydroponic *S. trifolia* in the ABA-treated and control groups, as well as the unexpanded stolon part, were collected. The fresh samples were quickly frozen with liquid nitrogen and stored in a −80 °C refrigerator for next use. Three biological replicates were conducted for each group.

The total RNA of stored samples was extracted using the Plant RNA Kit R6827 (Omega Bio-Tek, Norcross, GA, USA) according to the instructions. RNA concentration was detected using a NanoDrop One spectrophotometer (Thermo Fisher, Wilmington, DE, USA), and RNA quality was assessed using a Qubit 3.0 Fluorometer (Life Technologies, Carlsbad, CA, USA). After the RNA samples were qualified, the library was constructed using the NEBNext^®^ UltraTM RNA Library Prep Kit for Illumina^®^ (New England Biolabs, Beverly, MA, USA) kit. The constructed libraries were sequenced by an Illumina Novaseq 6000 (Illumina, San Diego, CA, USA) platform. The sequencing and library construction were completed by Benagen Technology Service Co., Ltd (Wuhan, China). The clean Reads were obtained from original Reads filtered by fastp (v0.21.0), and the quality control of the clean Reads was performed by using fastqc (v0.11.9).

### 4.4. Gene Function Annotation and DEGs Screening

Transcriptome analysis was performed on three types of samples, including expanded corms from the ABA-treated group (ABAexp), initially expanded corms from the untreated control group (CKexp), and unexpanded stolon tips from the untreated control group (CKunexp). Generated sequencing data were mapped to the unpublished assembled *S. trifolia* reference genome using StringTie (v2.1.4) software. The overall mapping rates of samples exceeded 97% (Table S3). Gene functional annotations were completed by aligning the Nr database. Gene expression abundance was quantified as fragments per kilobase of transcript per million mapped reads (FPKM). Differentially expressed genes (DEGs) between the comparison groups (ABAexp vs. CKexp, ABAexp vs. CKunexp, Ckexp vs. CKunexp) were identified using the DESeq2 (v1.26.0) R package [51]. Multiple-testing correction was conducted with the Benjamini–Hochberg false discovery rate (FDR) method to control false positives. Genes satisfying FDR < 0.05 and |log_2_ (fold change)| > 1 were defined as significantly differentially expressed genes, yielding DEGs involved in ABA-induced *S. trifolia* corm expansion under hydroponic conditions. Gene Ontology (GO) and KEGG enrichment were based on the clusterProfiler (v3.14.3) package [52]. In KEGG enrichment, the pathways with an adjusted *p*-value < 0.05 were regarded as significantly enriched.

### 4.5. Analysis of Expression Differences in Genes Related to Hormone Signal Transduction

The DEGs enriched in plant hormone signal transduction pathways in KEGG enrichment analysis were extracted from the comparison groups, including ABAexp vs. CKexp, ABAexp vs. CKunexp, and CKexp vs. CKunexp. The hormone signaling-related DEGs enriched in both the ABAexp vs. CKexp and ABAexp vs. CKunexp comparisons were integrated. Overlapping DEGs with the CKexp vs. CKunexp group were excluded to identify the DEGs associated with ABA-induced corm expansion in hydroponic conditions. The heat maps for visualizing gene expression were drawn using TBtools (v2.450) software [53].

### 4.6. Transcriptome Cross-Analysis and RT-qPCR Detection

Transcription factors (TFs) were identified from the screened DEGs related to the hormone signaling pathway associated with ABA-induced corm expansion in hydroponic *S. trifolia*. Based on the previously obtained transcriptome sequencing data of expanded corms of *S. trifolia* under sand-culture conditions and unexpanded stolon tips of *S. trifolia* under hydroponic conditions, the common differentially expressed transcription factors related to hormone signal transduction were identified by cross-analysis, and then uniformly named. TBtools software was used to draw the gene expression heatmap of each transcription factor, where the expression level of each gene was quantified by log_2_ (FPKM + 1).

RT-qPCR experiments were performed using the CFX96 Touch^TM^ Real-Time Quantitative PCR System (Bio-Rad, Hercules, CA, USA). The *StUBQ* gene from *S. trifolia* was selected as the internal control gene [54]. RT-qPCR was used to detect the expression levels of the target TF genes; the specific primers were designed with Primer Premier 6.0 and listed in Table 2. The PCR amplification results indicated that the primers used in the experiment have high amplification performance (Figure S1). Melting curve analysis was carried out following amplification to evaluate primer specificity; each target gene exhibited a single melting peak, suggesting specific amplification free of primer dimers (Figure S2). The total RT-qPCR reaction volume was 10 μL, comprising 4 μL diluted cDNA, 0.5 μL forward/reverse primers, and 5 μL iTaq Universal SYBR Green Supermix (Bio-Rad, Hercules, CA, USA). The reaction protocol was as follows: 3 min pre-denaturation at 95 °C and 39 cycles of 10 s denaturation at 95 °C, followed by 5 s annealing at 60 °C. Three biological replicates were set and each reaction was performed with three technical replicates. Relative gene expression was calculated using the 2^−ΔΔCt^ method [55].

### 4.7. Data Analysis

The mean and standard deviation of the data were calculated and analyzed using Excel 2019. The *t*-test analysis was performed using SPSS 20.0. The photograph was plotted using GraphPad Prism 8.0 software.

## Figures and Tables

**Table 1:** The DEGs identified from the transcriptome comparison.

Comparison	Significant_Diff_Number	Up	Down
CKexp_vs._ABAexp	2377	571	1806
CKunexp_vs._ABAexp	3692	1284	2408
CKunexp_vs._CKexp	2943	1274	1669

**Table 2:** The primers used for the RT-qPCR assay.

Gene ID	Forward/Reverse Primer Sequence (5′ → 3′)
*StriChr1G051240* (*EIN3*)	AGAAGCCGCATGACCTCAAGAAG/GGTTGATGACGGACAGCCAAGT
*StriChr3G133510* (*EIN3*)	AGCAGCAGCAGTTCAATCCAGAG/CGGAAGTCACCAGCGGCATT
*StriChr2G094080* (*ARF*)	GCAGAGGCAGAACTTGGTCAGTC/TGGCATCTGTGCTGGCATGTTAG
*StriChr5G184020* (*ARF*)	CGATAGGTCCAAGCAGCCAGTTC/GAGAGTCGCAGCCGAAGGATTG
*StriChr4G170530* (*ARF*)	GAGTGAGGAGAGTAGTGCGACAG/ATCAGGAGCCTCTTCACCTTCAA
*StriChr3G125830* (*ARR*)	GATGCCACAACTGACCATACCA/CGTGATGAACCAGCAGGACAAG

## Data Availability

The datasets analyzed and generated during the current study are available from the first author (008302@yzu.edu.cn) or corresponding author (ljli@yzu.edu.cn) on request.

## Supplementary Materials

The following supporting information can be downloaded at https://www.mdpi.com/article/10.3390/plants15131984/s1: Table S1: Statistics of sequencing data. Table S2: Identified candidate transcription factor genes. Table S3: Mapping rate of sequencing data against the reference genome. Figure S1: The PCR amplification results indicated that the primers used in the experiment have high amplification performance. Figure S2: Melting curves of primers used for RT-qPCR.

## Abbreviations

ABA, Abscisic acid; ARF, Auxin response factor; ARR, Arabidopsis response regulator; AUX/LAX, Auxin influx carrier; BRs, Brassinosteroids; BZR1, Brassinazole resistant 1; CK, Control; DEGs, Differentially expressed genes; EIN3, Ethylene insensitive 3; ETH, Ethylene; GA, Gibberellic acid; GO, Gene ontology; JA, Jasmonic acid; JAZ, Jasmonate ZIM-Domain protein; KEGG, Kyoto Encyclopedia of Genes and Genomes; MeJA, Methyl jasmonate; NPR1, Non-expressor of pathogenesis-related genes 1; RT-qPCR, Real-time quantitative PCR; SA, Salicylic acid; SNRK2, Sucrose non-fermenting 1-related orotein kinase 2; vs., versus.
