# Supplementary material for: Exogenous Hormones Affect the Corm Expansion of Sagittaria trifolia in Hydroponic Conditions
Source: Plants (Basel). 2026 Jun 26;15(13):1984. doi: 10.3390/plants15131984 (PMC13364254; doi:10.3390/plants15131984)
Supplement: Supplementary file 1 [file plants-15-01984-s001.zip › plants-4368066-supplementary.pdf]

Table S1 Statistics of sequencing data.

Sample	Clean reads	Clean bases	Q 20 Ratio	Q 30 Ratio	GC content
ABAexp-1	43,126,938	6,344,349,186	99%	95%	51%
ABAcxp-2	39,400,308	5,807,995,937	98%	95%	51%
ABAexp-3	43,083,592	6,359,811,819	98%	95%	51%
CKexp-1	43,042,280	6,258,719,225	98%	95%	51%
CKexp-2	43,022,968	6,282,381,693	98%	95%	51%
CKexp-3	43,053,210	6,305,086,534	98%	95%	51%
CKunexp-1	39,749,464	5,843,244,008	98%	95%	51%
CKunexp-2	43,050,260	6,336,389,017	98 %	95%	51%
CKunexp-3	43,026,308	6,309,600,494	98 %	95%	51%

Table S2 Identified candidate transcription factor genes.

Transcript ID	PlantTFDB annotation	Name
StriChrIG051240.1	EIN3	*StriChr1G051240* (*EIN3*)
StriChr3G133510.1	EIN3	*StriChr3G133510* (*EIN3*)
StriChr2G094080.t1	ARF	*StriChr2G094080* (*ARF*)
StriChr3G125830.t5	ARR	*StriChr3G125830* (*ARR*)
StriChr4G170530.1	ARF	*StriChr4G170530* (*ARF*)
StriChr5G184020.1	ARF	*StriChr5G184020* (*ARF*)

Table S3 Mapping rate of sequencing data against the reference genome.

Sample	Total reads(bp)	Mapping rate (%)
ABAexp1	21,563,469	98.06
ABAexp2	19,700,154	97.87
ABAexp3	21,541,796	97.91
CKexp1	21,521,140	98.08
CKexp2	21,511,484	97.82
CKexp3	21,526,605	97.76
CKunexp1	19,874,732	97.66
CKunexp2	21,525,130	97.49
CKunexp3	21,513,154	97.7

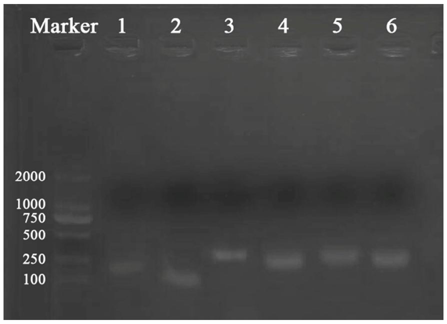

Figure S1 The PCR amplification results indicated that the primers used in the experiment have high amplification performance.

(1～6) represent the PCR amplification of primers for *StriChr1G051240* (*EIN3*); *StriChr3G133510* (*EIN3*); *StriChr2G094080* (*ARF*); *StriChr5G184020* (*ARF*), *StriChr4G170530* (*ARF*) and *StriChr3G125830* (*ARR*), respectively.

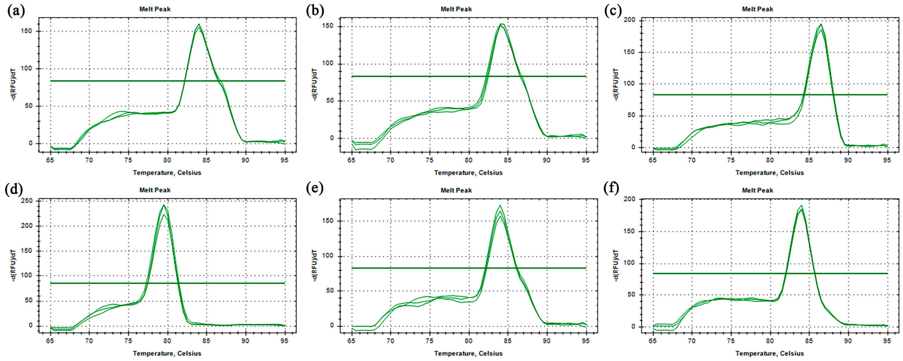

Figure S2 Melting curves of primers used for RT-qPCR.

(a) *StriChr1G051240* (*EIN3*); (b) *StriChr3G133510* (*EIN3*); (c) *StriChr2G094080* (*ARF*); (d) *StriChr5G184020* (*ARF*) (e) *StriChr4G170530*(*ARF*) (f) *StriChr3G125830* (*ARR*)
